# Supplementary material for: OpenMRF: A Modular, Vendor‐Neutral Open‐Source Framework for Magnetic Resonance Fingerprinting Using Pulseq
Source: Magn Reson Med. 2026 Sep 6;97(1):114–32. doi: 10.1002/mrm.70586 (PMC13746074; doi:10.1002/mrm.70586)
Supplement: Supplementary file 1 [file MRM-97-114-s001.pdf]

Supplementary Material

OpenMRF: A Modular, Vendor-Neutral Open-Source Framework for Magnetic Resonance Fingerprinting using Pulseq

S1 OpenMRF GitHub overview

OpenMRF is organized as a GitHub organization (github.com/OpenMRF) containing multiple repositories. The organization separates the main framework source code, project documentation, and publication-specific resources to facilitate long-term maintenance and ensure reproducibility.

```
OpenMRF (GitHub Organization; https://github.com/OpenMRF)
│
├── openmrf-documentation/       → openmrf.org: website & documentation
│   ├── MkDocs source code
│   ├── docs/                    → documentation pages
│   │   ├── wiki/                → wikipedia: prep. modules, readout modules…
│   │   ├── blog.md              → development news
│   │   ├── index.md             → homepage
│   │   ├── publications.md      → OpenMRF publications
│   │   ├── quickstart.md        → installation guide
│   │   ├── team.md              → project contributors
│   │   └── troubleshoot.md      → FAQ & troubleshooting
│   └── .github/                 → website deployment workflows
│
├── openmrf-publications/        → publication-specific resources
│   ├── 2026_MRM_OpenMRF/        → resources accompanying the present study
│   │   ├── Pulseq/              → Pulseq sequence definitions
│   │   ├── Reconstructions/     → reconstruction including dictionary generation
│   │   └── Simulations/         → numerical phantom simulations
│   ├── YYYY_Journal_A/
│   ├── YYYY_Journal_B/
│   └── ...
│
├── openmrf-core-matlab/         → MATLAB framework
│   ├── include_cwru/            → CWRU specific source code and EULA
│   ├── include_miitt/           → MIRT and low-rank reconstruction
│   ├── include_misc/            → utility functions
│   ├── include_pre_sim_library/ → precomputed adiabatic pulses and slice profiles
│   ├── include_pulseq_master/   → Original Pulseq v1.5.1
│   ├── include_pulseq_toolbox/  → OpenMRF specific Pulseq extensions
│   ├── main_sequences/          → sequence examples and reconstruction pipelines
│   ├── user_specifications/     → user and scanner configurations
│   ├── install_OpenMRF.m        → framework installation script
│   ├── LICENSE                  → MIT license
│   ├── NOTICE                   → third-party software and licenses
│   └── README.md                → project overview
│
└── openmrf-core-python/         → future Python based release
```

The ***openmrf-documentation*** repository contains the source code of the ***openmrf.org*** website, which provides installation instructions, tutorials, troubleshooting information, and a project wiki describing the modular sequence architecture of OpenMRF.

The ***openmrf-publications*** repository contains publication-specific resources. Current and future publications are assigned a dedicated directory containing the Pulseq sequence code, numerical simulations, reconstruction scripts, and all configuration files required to reproduce the corresponding results. For the present study, these resources are provided in the folder ***2026_MRM_OpenMRF***.

The ***openmrf-core-matlab*** repository contains the MATLAB implementation of OpenMRF, including the original Pulseq v1.5.1 source code, OpenMRF-specific Pulseq extensions, simulation pipelines, reconstruction pipelines, utility functions, precomputed simulation libraries, and user/hardware configurations.

A native Python implementation (***openmrf-core-python***) is currently under development and will provide equivalent functionality in a Python-based environment.

S2 Third-party software contributions

OpenMRF integrates several publicly available software packages that provide specialized functionality while keeping their source code clearly separated from the primary OpenMRF implementation. Unless explicitly stated otherwise, all source code was developed as part of the OpenMRF project. External software components are included in dedicated repository subdirectories and retain their original licenses, copyrights, and citations. The corresponding information is summarized in the repository ***NOTICE*** file.

The primary external dependency is the original Pulseq framework (***include_pulseq_master***), which provides the vendor-neutral sequence definition standard on which OpenMRF is built. OpenMRF extends the original Pulseq implementation by providing additional sequence modules, simulation tools, reconstruction pipelines, and hardware-specific utilities (***include_pulseq_toolbox***) while preserving compatibility with the original Pulseq framework.

Image reconstruction relies on two external software components contained in ***include_miitt***. The Michigan Image Reconstruction Toolbox (MIRT) provides the underlying non-cartesian reconstruction algorithms, while the low-rank reconstruction implementation developed at the Michigan Institute for Imaging Technology and Translation (MIITT) provides the MRF-specific reconstruction framework used throughout this work.

The directory ***include_cwru*** contains MRF-specific source code distributed by Case Western Reserve University (CWRU) under a separate End User License Agreement (EULA). This component remains the intellectual property of CWRU and is included under the conditions specified in the accompanying EULA.

Additional third-party software is incorporated as independent utility modules within ***include_misc***, organized into dedicated subdirectories. These include scientific color maps for visualization of relaxation time maps (*src_colormaps*), as well as the Eigen C++ library for linear algebra operations used in MEX-based EPG and Bloch simulations (*src_eigen*). Coil sensitivity estimation is implemented via ESPIRiT (*src_espirit*), while Siemens raw data import is enabled through the mapVBVD toolbox (*src_mapVBVD*). MRI phantom simulation capabilities are provided using realistic coil sensitivity maps (*src_mri_phantom*). Additional modules include peripheral nerve stimulation prediction using the SAFE model (*src_safe*), time-optimized gradient waveform design (*src_tOptGrad*), and variable-density spiral trajectory design (*src_vds*).

S3 Dictionary parameters

Dictionary generation was performed automatically from the corresponding Pulseq sequence definitions as described in the Methods section. Depending on the application, different parameter spaces were used for dictionary generation. At 3 T, transmit field inhomogeneity (B_1^+) was included as an additional matching parameter. For the T_1-T_2-$T_{1\rho}$ cMRF sequence, $T_{1\rho}$ was incorporated as a third relaxation parameter. Off-resonance effects (ΔB_0) were not included in the dictionaries and were fixed to $\Delta B_0 = 0$ for all experiments.

The discrete dictionary values were generated using exponentially increasing sampling (multiplicative spacing) for relaxation parameters and linear sampling for B_1^+. The corresponding parameter ranges and discretization are summarized in Table S1.

Supplementary Table S1. Dictionary parameters used for the MRF experiments presented in this work.

	MRF & cMRF (0.55 T / 1.5 T)	MRF & cMRF (3 T)	T_1-T_2-$T_{1\rho}$ cMRF
T_1	10 ms – 4 s (×1.025)	10 ms – 4 s (×1.025)	10 ms – 4 s (×1.05)
T_2	1 ms – 3 s (×1.025)	1 ms – 3 s (×1.025)	1 ms – 3 s (×1.05)
$T_{1\rho}$	—	—	1 ms – 3 s (×1.05)
B_1^+	fixed to 1	0.80–1.20 (step 0.025)	fixed to 1
**Physical constraints**	$T_2 < T_1$	$T_2 < T_1$	$T_2 < T_1$, $T_{1\rho} < T_1$, $T_2 < T_{1\rho}$
**Dictionary entries**	**52,179**	**887,043**	**799,014**

S4 T_1, T_2 and $T_{1\rho}$ reference protocols

Reference measurements were acquired independently at each participating site and field strength to account for differences between individual ISMRM/NIST phantom versions and on-site conditions (e.g. temperature). T_1 and T_2 reference values were obtained using conventional inversion-recovery spin-echo and spin-echo sequences, respectively. $T_{1\rho}$ reference values were measured using a dedicated Pulseq-based single-parameter mapping sequence employing the same spin-lock preparation module as the corresponding T_1-T_2-$T_{1\rho}$ cMRF acquisition. The sequence parameters used for the reference measurements are summarized below.

T_1 reference protocol: T_1 reference measurements were performed using a vendor-provided 2D inversion-recovery spin-echo sequence. A single coronal slice (6 mm thickness) was acquired in the $MnCl_2$ layer of the ISMRM/NIST phantom with an in-plane resolution of 1.95 × 1.95 mm^2 (FOV = 250 × 250 mm^2, matrix = 128 × 128). The repetition time was fixed to 5 s and the echo time was minimized (TE = 6.4 ms). Ten measurements were acquired using inversion times of 35, 60, 95, 160, 260, 430, 710, 1180, 1940, and 3200 ms, while all other imaging parameters were kept constant. The total duration of the T_1 reference protocol was approximately 1 hour and 47 minutes. Reference T_1 values were subsequently estimated by voxel-wise fitting of the inversion-recovery signal model.

T_2 reference protocol: T_2 reference measurements were performed using the same vendor-provided 2D spin-echo sequence without inversion preparation. The imaging geometry and acquisition parameters were identical to the T_1 reference protocol, including a single coronal slice (6 mm thickness), an in-plane resolution of 1.95 × 1.95 mm^2 (FOV = 250 × 250 mm^2, matrix = 128 × 128), and a repetition time of 5 s. Ten measurements were acquired using echo times of 10, 15, 20, 30, 40, 60, 85, 120, 175, and 250 ms, while all other imaging parameters were kept constant. The total duration of the T_2 reference protocol was approximately 1 hour and 47 minutes. Reference T_2 values were subsequently estimated by voxel-wise fitting of a mono-exponential signal decay model.

$T_{1\rho}$ reference protocol: $T_{1\rho}$ reference measurements were performed using a dedicated Pulseq-based 2D spiral mapping sequence implemented in OpenMRF. A single slice was acquired with FOV = 300 × 300 mm^2, matrix size = 192 × 192, and slice thickness = 8 mm. Each $T_{1\rho}$-weighted image was acquired using eight spiral readouts with an Equal-2π interleaf ordering. Before each preparation, a saturation module was applied followed by a 5 s recovery delay. $T_{1\rho}$ preparation was performed using the same balanced spin-lock module as implemented in the T_1-T_2-$T_{1\rho}$ cMRF sequence, with adiabatic half-passage pulses, a spin-lock amplitude of 300 Hz (100 Hz for 0.55 T), and spin-lock durations of 8, 16, 24, 32, 40, 48, 56, 64, 72, and 80 ms. The total duration of the $T_{1\rho}$ reference protocol was approximately 6.8 minutes. Reference $T_{1\rho}$ values were subsequently estimated by voxel-wise fitting of a mono-exponential signal decay model.

Supplementary Table S2. Reference T_1, T_2 and $T_{1\rho}$ values (ms) measured independently at each participating site and field strength using the MRI protocol described in the previous section. Missing values were not included in the quantitative analysis because the corresponding phantom inserts exhibited low fitting accuracy, while insert #14 was additionally excluded due to manufacturing-related inconsistencies observed between different ISMRM/NIST phantom versions.

T_1 reference values

B_0	0.55 T	1.5 T	1.5 T	1.5 T	3.0 T	3.0 T	3.0 T	3.0 T
Vendor	Siemens	Siemens	Siemens	Siemens	Siemens	Siemens	Siemens	UI
Platform	Free.Max	Aera	Avanto	Sola	Cima.X	Prisma	Vida	uMR790
Site	Ann Arbor	Freiburg	Würzburg	Ann Arbor	Freiburg	Würzburg	Ann Arbor	Houston
#1	---	---	---	---	---	---	---	---
#2	92.8	115	123	120	126	128	119	127
#3	126	159	164	158	172	171	154	174
#4	179	225	231	218	243	241	217	245
#5	231	289	297	274	312	310	279	315
#6	346	427	435	408	459	453	411	465
#7	459	599	613	546	643	637	547	650
#8	600	801	808	702	859	843	716	860
#9	790	1014	1000	908	1084	1041	928	1073
#10	1018	1512	1149	1177	1613	1197	1170	1541
#11	1241	1559	1480	1400	1665	1548	1407	1583
#12	1519	1924	1830	1697	2046	1866	1710	1896
#13	1756	2256	2145	1936	2372	2152	1933	2163
#14	---	---	---	---	---	---	---	---

T_2 reference values

B_0	0.55 T	1.5 T	1.5 T	1.5 T	3.0 T	3.0 T	3.0 T	3.0 T
Vendor	Siemens	Siemens	Siemens	Siemens	Siemens	Siemens	Siemens	UI
Platform	Free.Max	Aera	Avanto	Sola	Cima.X	Prisma	Vida	uMR790
Site	Ann Arbor	Freiburg	Würzburg	Ann Arbor	Freiburg	Würzburg	Ann Arbor	Houston
#1	---	---	---	---	---	---	---	---
#2	19.1	11.3	12.2	13.1	7.9	8.4	9.5	7.8
#3	25.8	15.7	16.1	16.9	11.0	11.1	11.4	10.6
#4	38.1	22.8	23.1	23.4	15.8	15.9	16.1	15.2
#5	49.8	29.8	30.5	29.8	20.6	20.7	20.7	19.9
#6	78.3	45.7	46.8	47.1	31.6	32.0	31.7	30.8
#7	108	67.9	70.7	66.9	47.0	47.6	44.7	45.9
#8	149	98.3	101	89.7	66.8	68.6	61.8	65.7
#9	216	135	136	131	91.8	92.9	88.8	90.3
#10	308	247	168	181	172	115	125	170
#11	415	260	255	252	181	178	173	179
#12	580	387	374	379	275	263	258	270
#13	798	556	571	536	399	406	372	382
#14	---	---	---	---	---	---	---	---

$T_{1\rho}$ reference values

B_0	0.55 T	1.5 T	1.5 T	1.5 T	3.0 T	3.0 T	3.0 T	3.0 T
Vendor	Siemens	Siemens	Siemens	Siemens	Siemens	Siemens	Siemens	UI
Platform	Free.Max	Aera	Avanto	Sola	Cima.X	Prisma	Vida	uMR790
Site	Ann Arbor	Freiburg	Würzburg	Ann Arbor	Freiburg	Würzburg	Ann Arbor	Houston
#1	---	---	---	---	---	---	---	---
#2	---	---	---	---	---	---	---	---
#3	---	---	---	---	---	---	---	---
#4	---	---	---	---	---	---	---	---
#5	---	---	---	---	---	---	---	---
#6	95.3	51.1	47.5	56.8	36.7	43.0	34.9	---
#7	129	69.4	69.8	69.0	50.6	56.8	45.5	---
#8	169	96.2	103	97.2	68.8	73.4	65.8	---
#9	236	132	140	136	95.1	94.6	92.2	---
#10	320	248	171	196	178	121	129	---
#11	453	262	263	271	201	189	182	---
#12	642	384	385	412	298	276	278	---
#13	917	569	574	571	442	418	419	---
#14	---	---	---	---	---	---	---	---

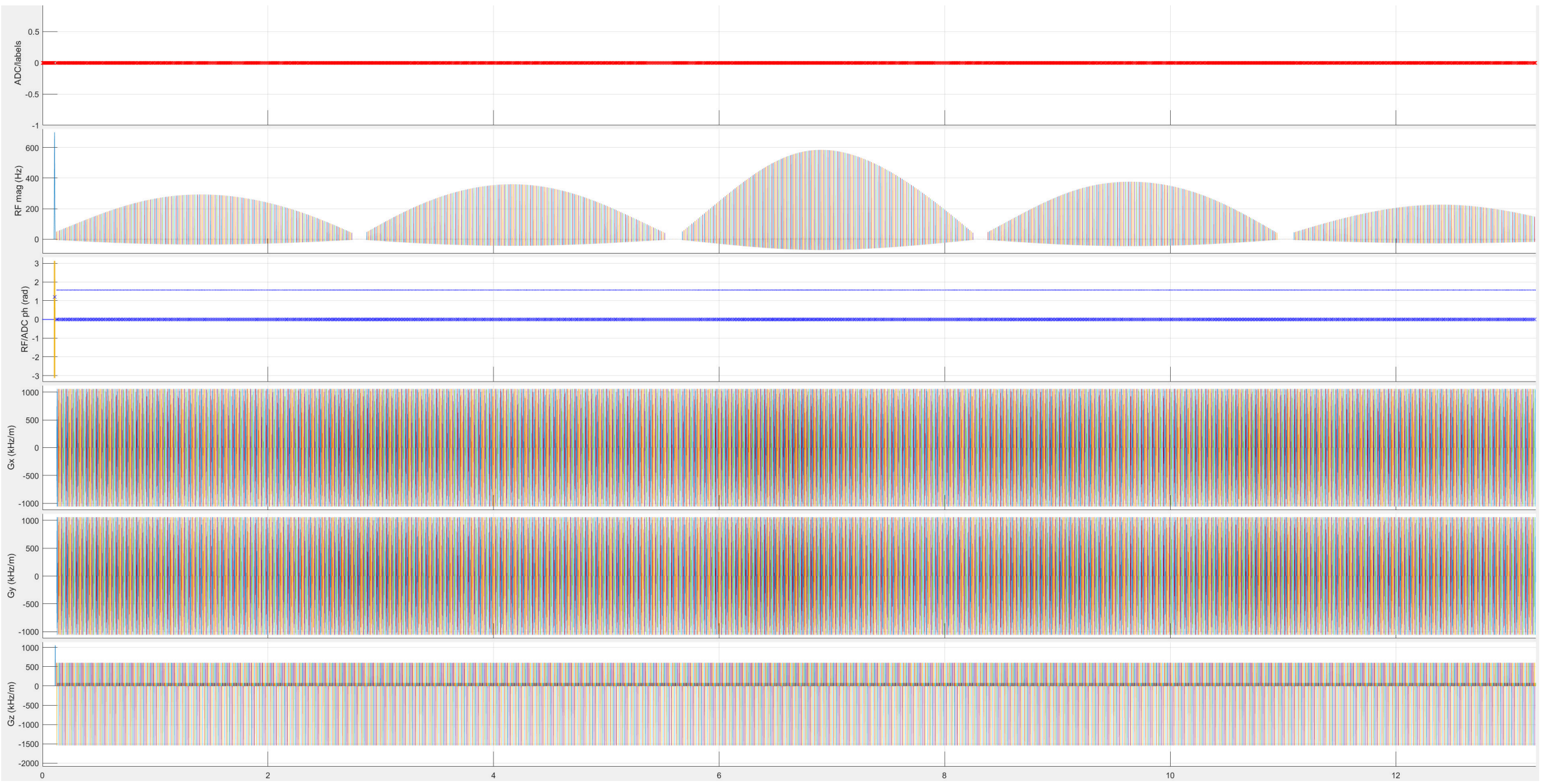

Supplementary Figure S1) Pulseq sequence diagram of the IR-FISP MRF sequence. The implemented method is based on variable flip angles and repetition times following an adiabatic inversion.

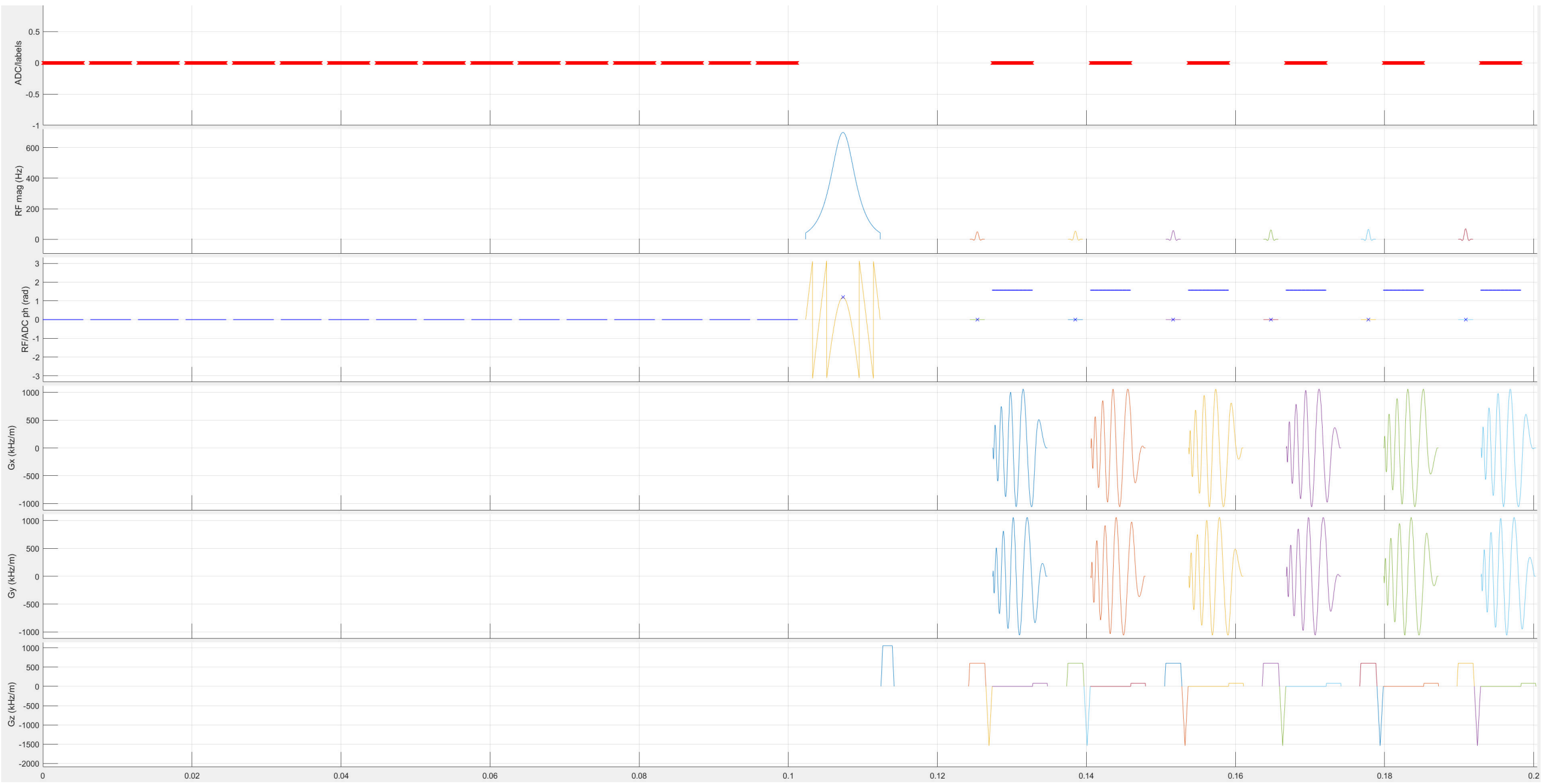

Supplementary Figure S2) Enlarged section of the IR-FISP MRF sequence. The acquisition starts with multiple noise pre-scans, while the actual MRF sequence begins with an adiabatic inversion pulse (hyperbolic secant). The figure also depicts the first six spiral readouts.

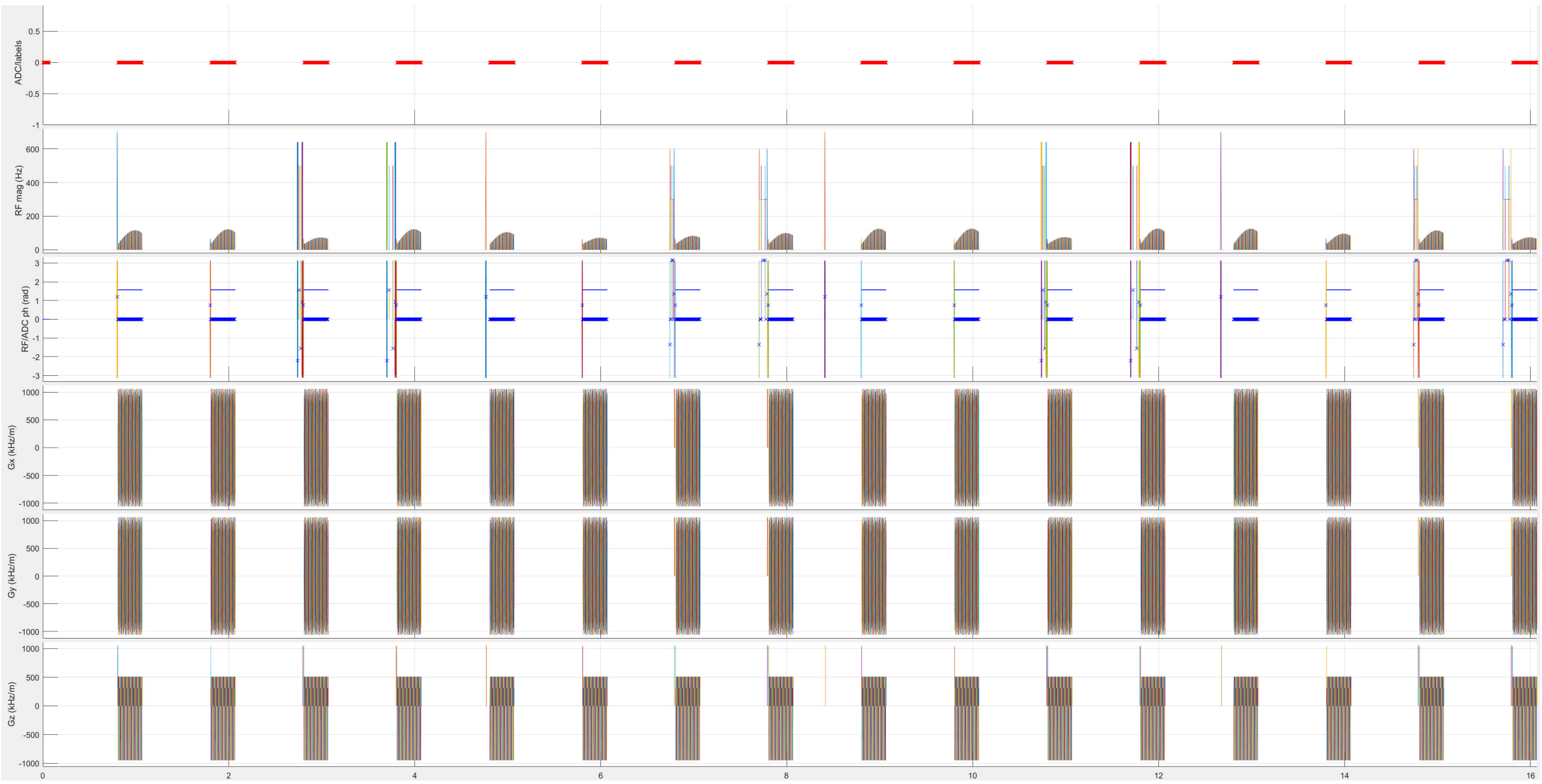

Supplementary Figure S3) Pulseq sequence diagram of the T_1-T_2-$T_{1\rho}$ cMRF sequence. The method was used for abdominal imaging, or for the heart by combining the protocol with prospective triggering. The sequence consists of 16 segments with different preparation modules, each followed by 48 spiral readouts.

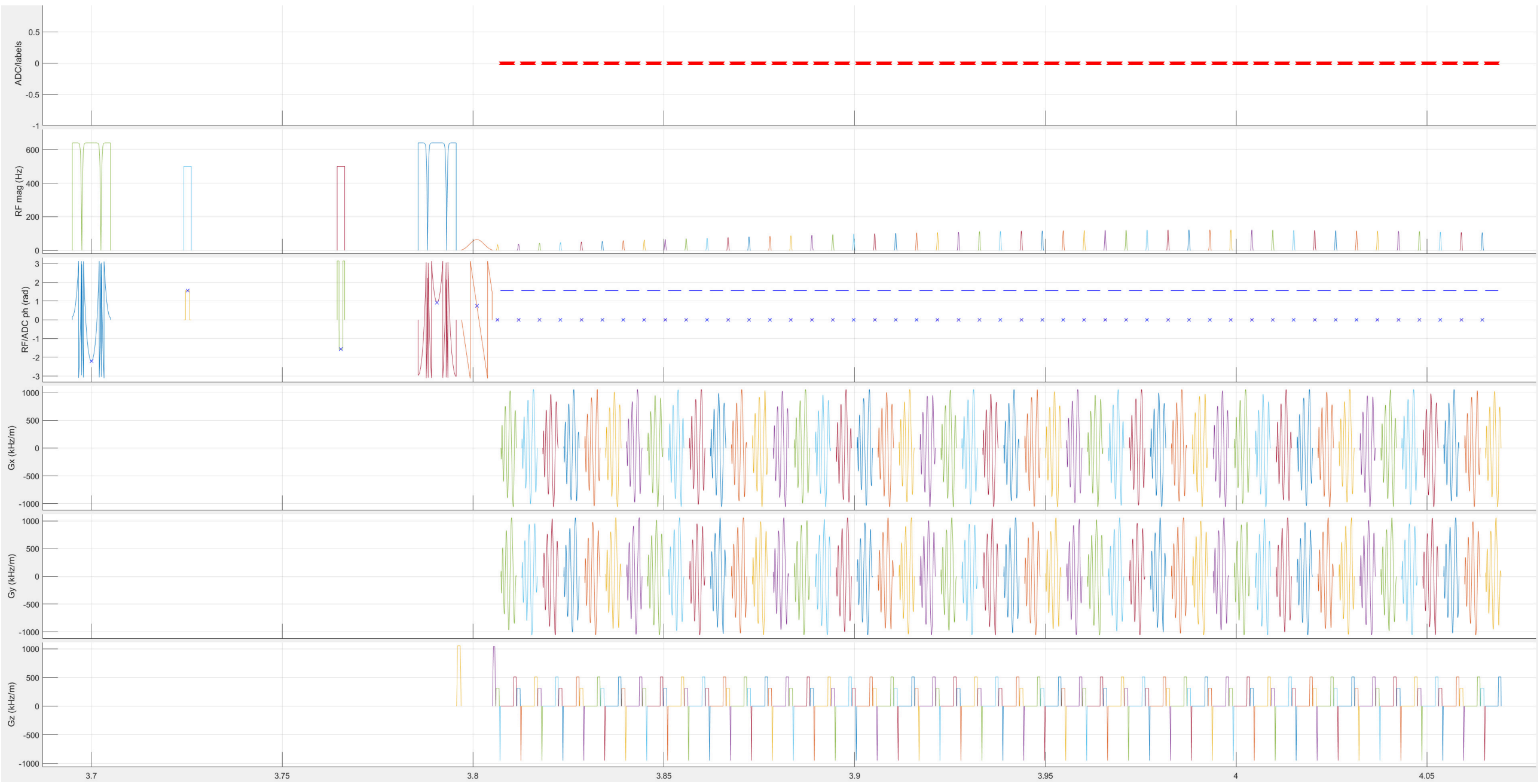

Supplementary Figure S4) Sequence diagram of the T_2 preparation module used in the cMRF sequences. The preparation utilizes adiabatic excitation (BIR-4) and composite refocusing pulses for improved B_0 and B_1^+ robustness.

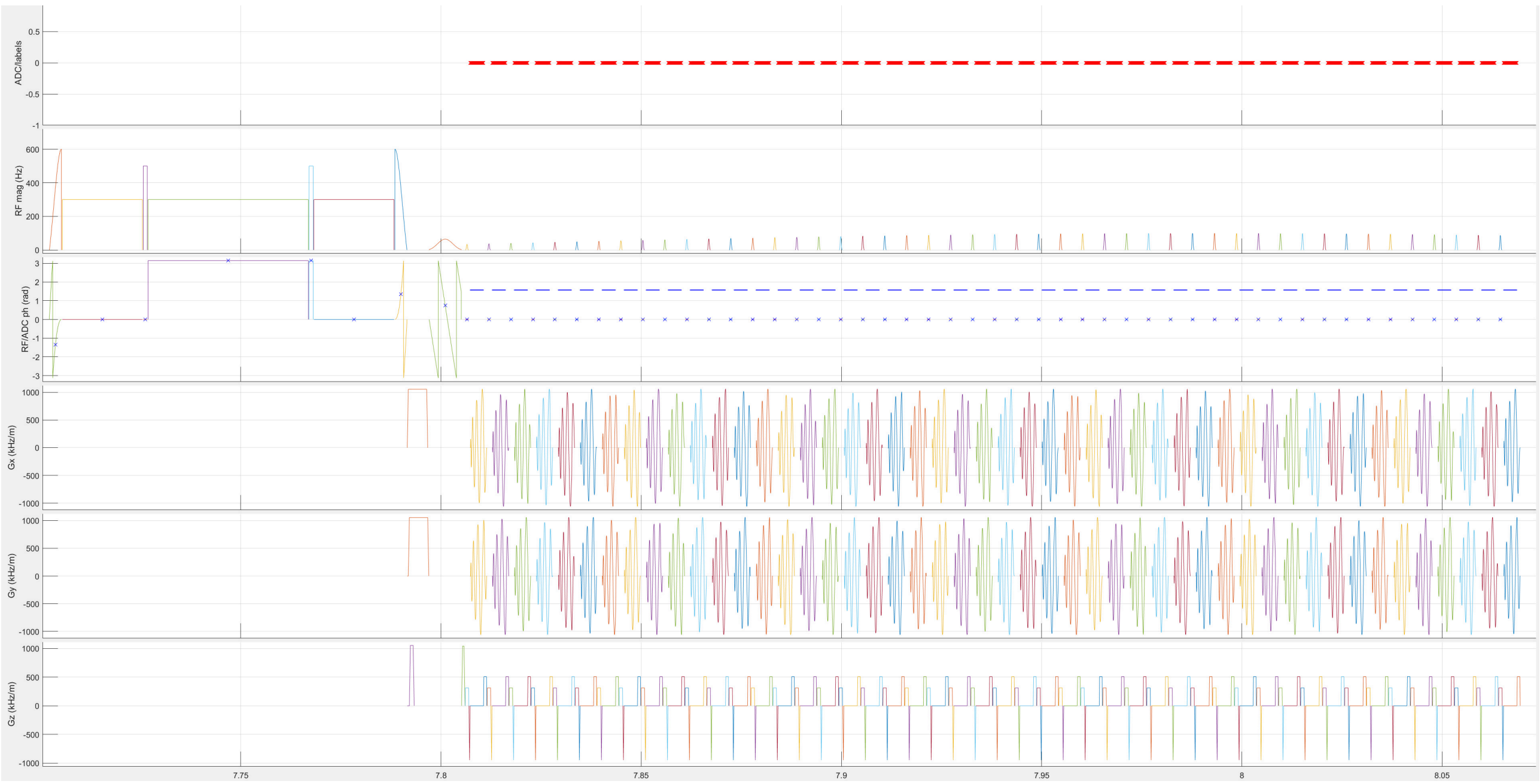

Supplementary Figure S5) Sequence diagram of the $T_{1\rho}$ preparation module used in the cMRF sequences. The preparation utilizes adiabatic half passage excitation pulses and rotary-echo phase cycling based on the balanced spin-lock concept for improved B_0 and B_1^+ robustness.

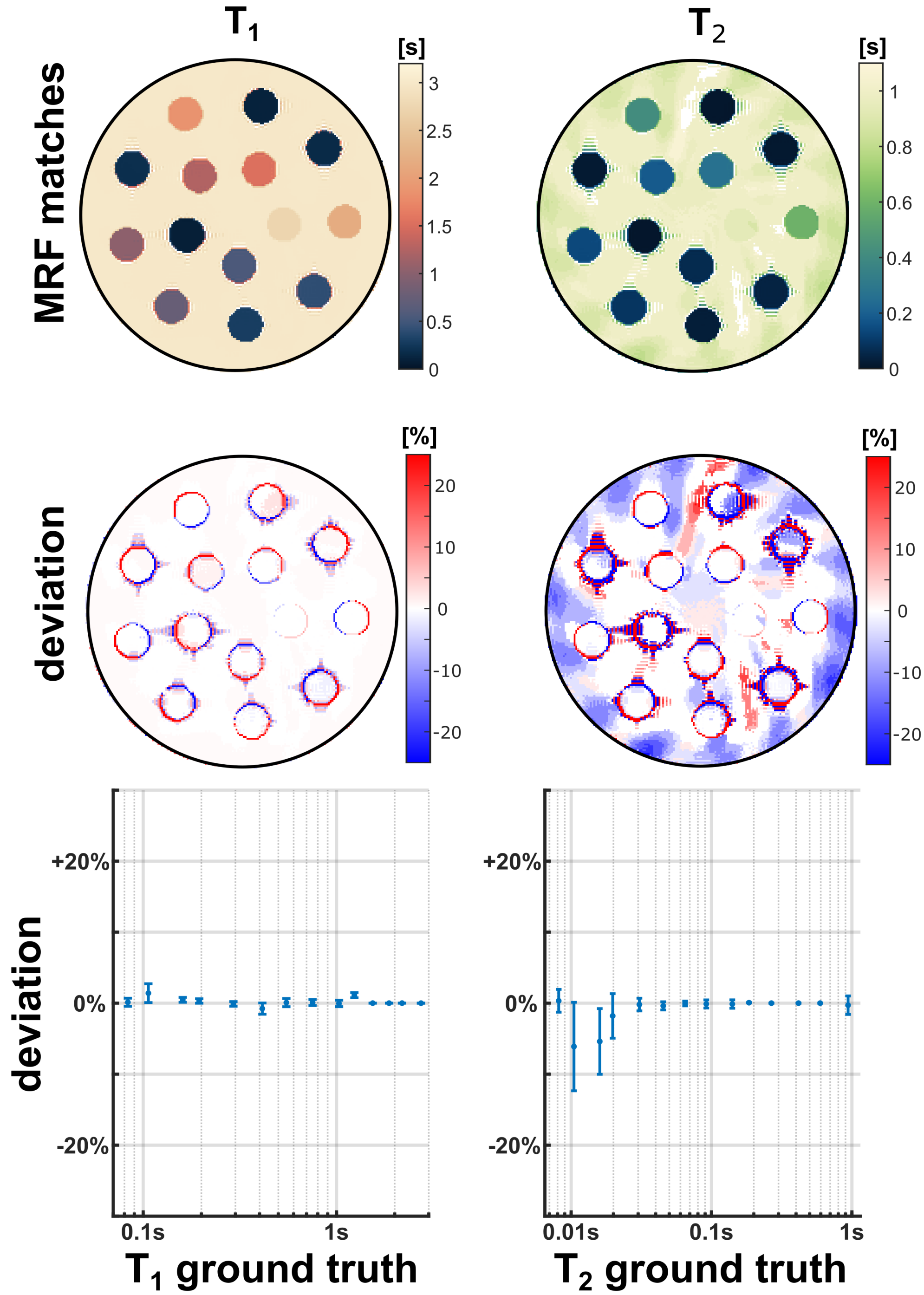

Supplementary Figure S6) Digital phantom simulation of the T_1-T_2-cMRF sequence. The reconstruction was carried out using the low-rank approach. The estimated parameters are shown in the upper row. Deviations from the ground truth are depicted in the middle row and the deviations evaluated inside the individual inserts are shown in the bottom row. T_2 shows increased deviations in the phantom background and in inserts with short and long relaxation times. The mean deviations in the contrast spheres are 0.19$\pm$0.74 % (T_1) and -1.08$\pm$3.07 % (T_2).

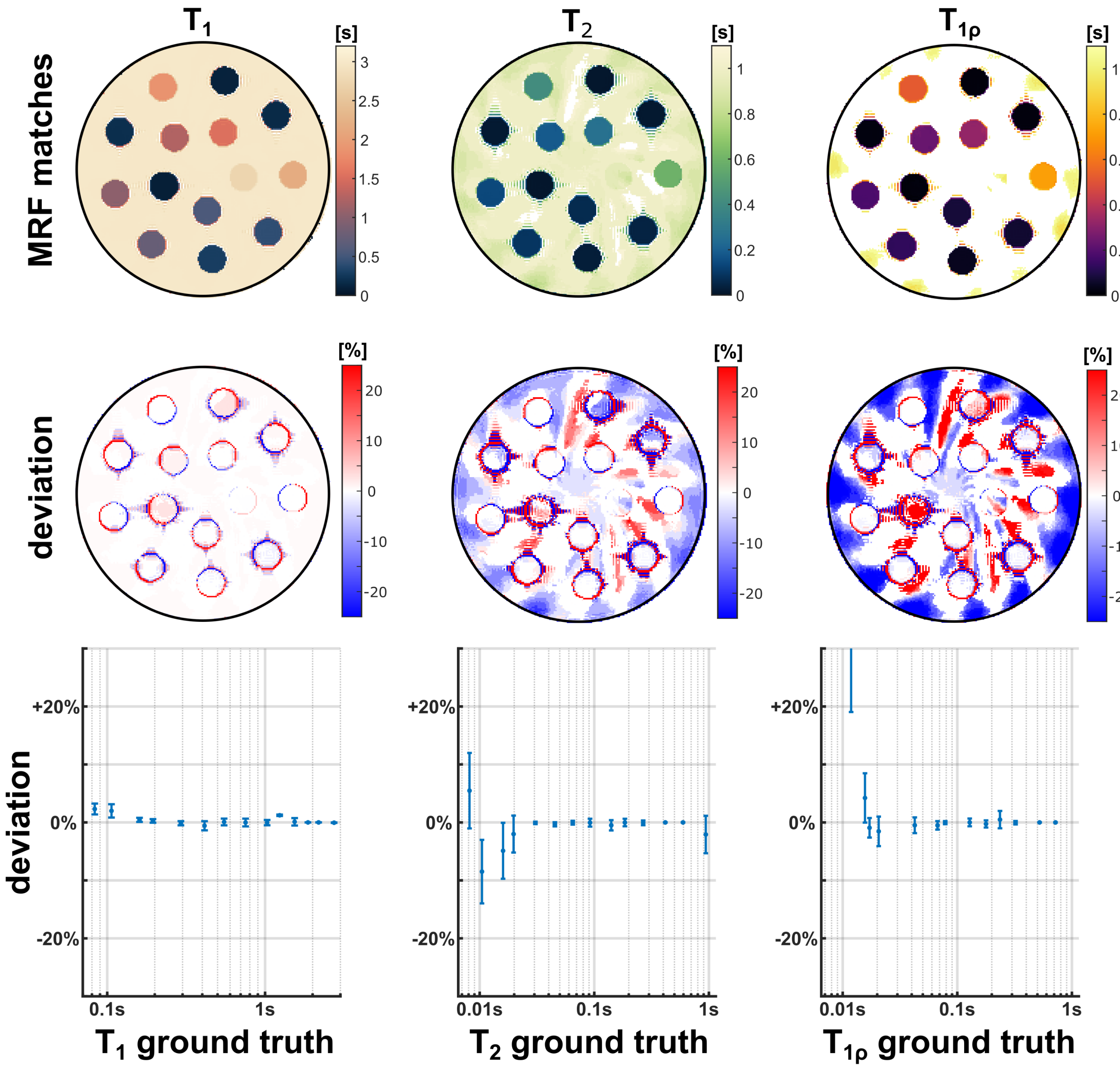

Supplementary Figure S7) Digital phantom simulation of the T_1-T_2-$T_{1\rho}$-cMRF sequence. The reconstruction was carried out using the low-rank approach. The estimated parameters are shown in the upper row. Deviations from the ground truth are depicted in the middle row and the deviations evaluated inside the individual inserts are shown in the bottom row. T_2 and $T_{1\rho}$ show increased deviations in the phantom background and in inserts with short and long relaxation times. The mean deviations in the contrast spheres are 0.40±0.99 % (T_1), -0.94±4.15 % (T_2) and -0.10±0.84% ($T_{1\rho}$).

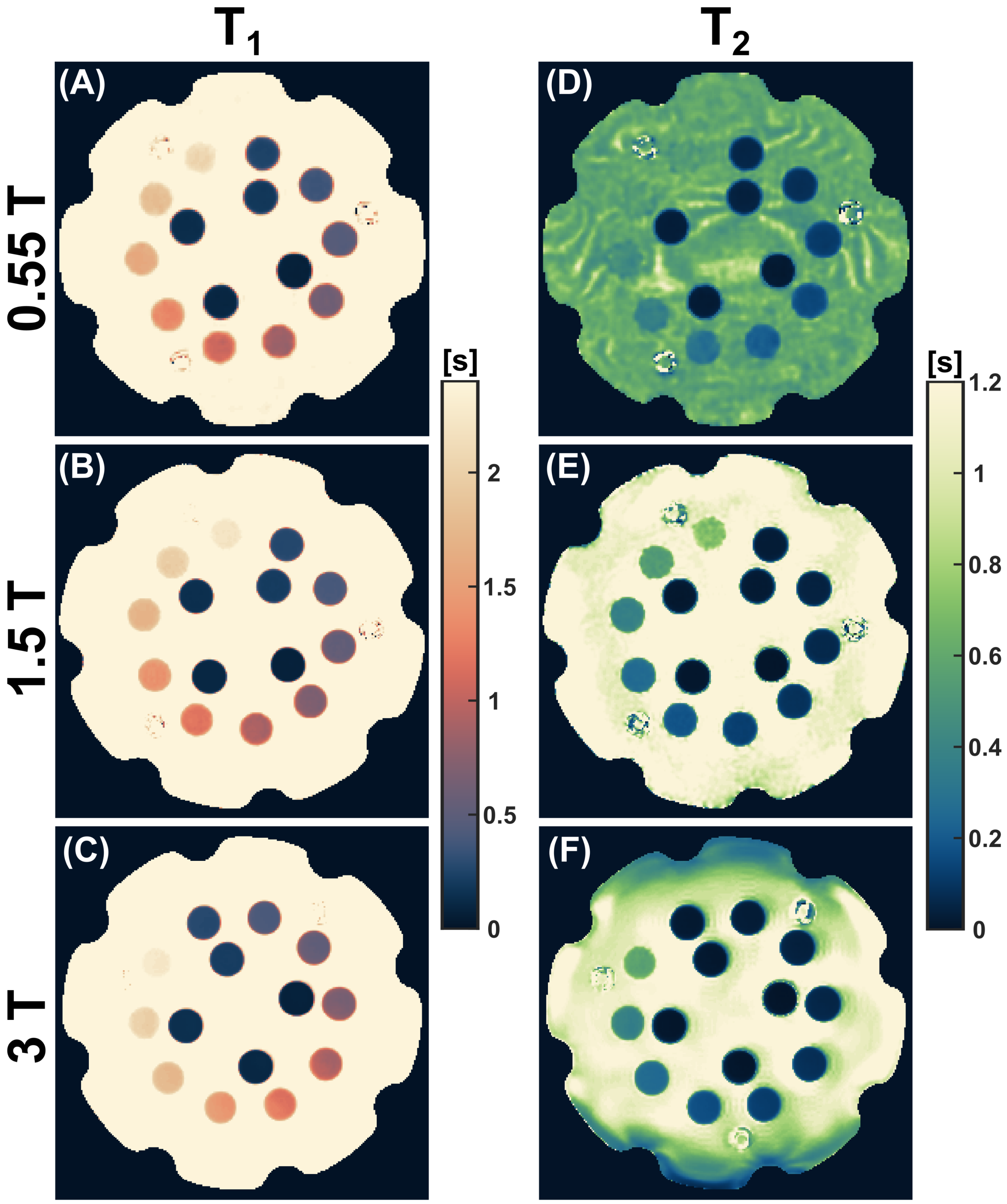

Supplementary Figure S8) Multi–field-strength validation of the T_1-T_2 cardiac MRF (cMRF) sequence in the ISMRM/NIST system phantom, acquired on Siemens Free.Max (0.55 T), Sola (1.5 T), and Vida (3 T) scanners. All datasets were reconstructed using the low-rank subspace method. The dictionary was generated with T_1 values ranging from 10 ms to 4 s in 2.5% steps, and T_2 values ranging from 1 ms to 3 s in 2.5% steps. Only physically relevant combinations were used ($T_2 < T_1$). A–C) Reconstructed T_1 maps and D–F) T_2 maps at 0.55 T, 1.5 T, and 3 T, respectively. Simultaneous estimation of both relaxation parameters was successfully achieved.

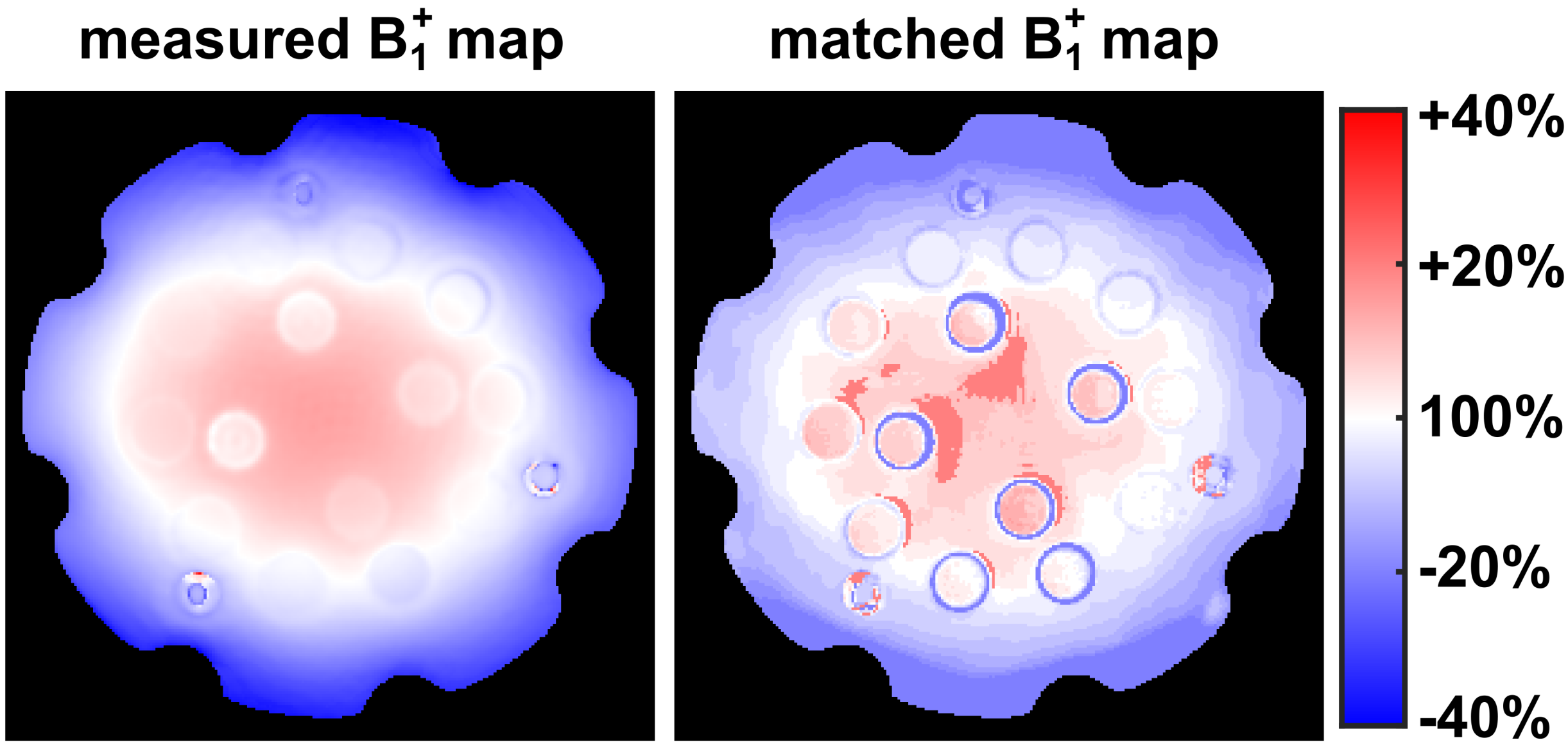

Supplementary Figure S9) Comparison of a reference B_1^+ map obtained with the WASABI method and a matched B_1^+ map estimated using the T_1-T_2-cMRF sequence. Data were acquired in the ISMRM/NIST phantom on a 3T Siemens Prisma scanner. The MRF-derived B_1^+ map closely matches the spatial structure of the reference, capturing the characteristic central brightening associated with increased RF transmission in the phantom center. The B_1^+ effect in the MRF dictionary was modeled on a coarse grid with 2.5% increments. Notably, the WASABI reference exhibits reduced B_1^+ values in inserts with short relaxation times, caused by relaxation during the WASABI preparation pulse, which was not accounted for in the fit routine. For these inserts, the MRF-estimated B_1^+ values can therefore be considered more accurate.

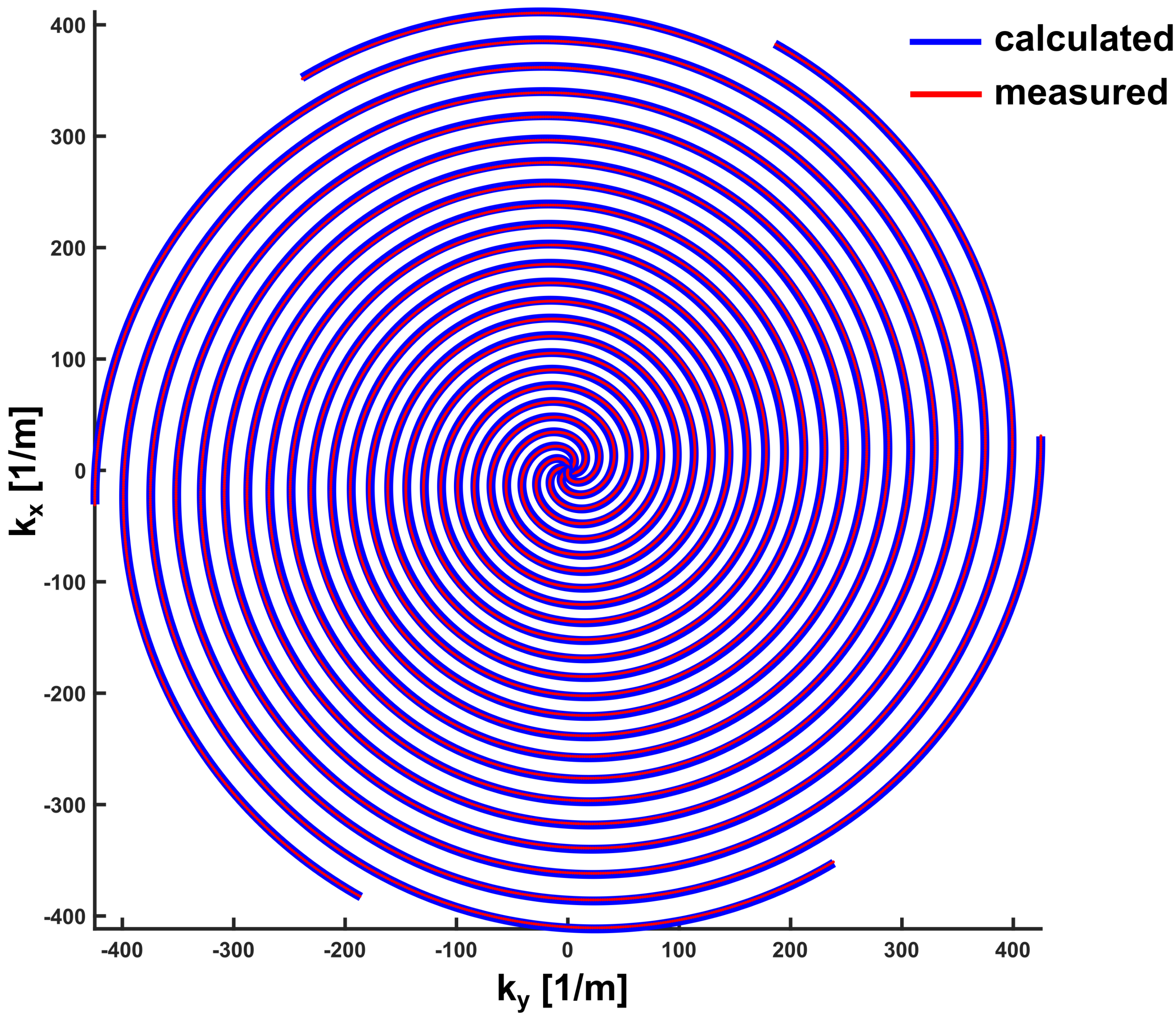

Supplementary Figure S10) Comparison of the calculated and measured k-space trajectory for the IR-FISP MRF sequence for 6 spiral interleaves. The calculation of the k-space trajectory was achieved using Pulseq's default tools. The measurement was carried out using the approach described by Robison et al. in a spherical calibration phantom. The .seq file for this calibration step can be generated automatically using OpenMRF.

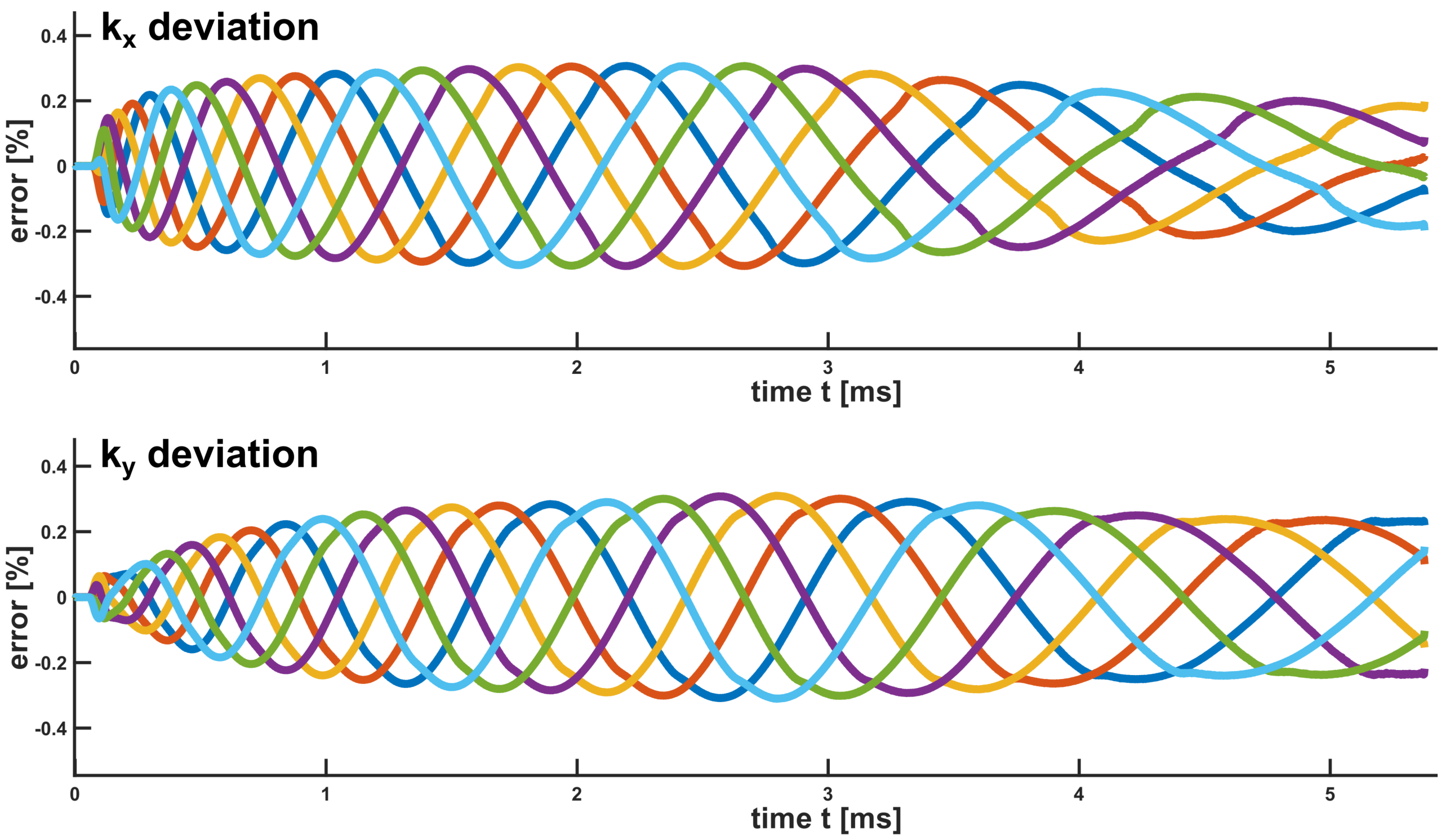

Supplementary Figure S11) Deviation of the measured k-space trajectory from the calculated reference. The deviation is shown as the relative error compared to the nominal $k_{\max}$ of the entire trajectory (426.67 1/m).

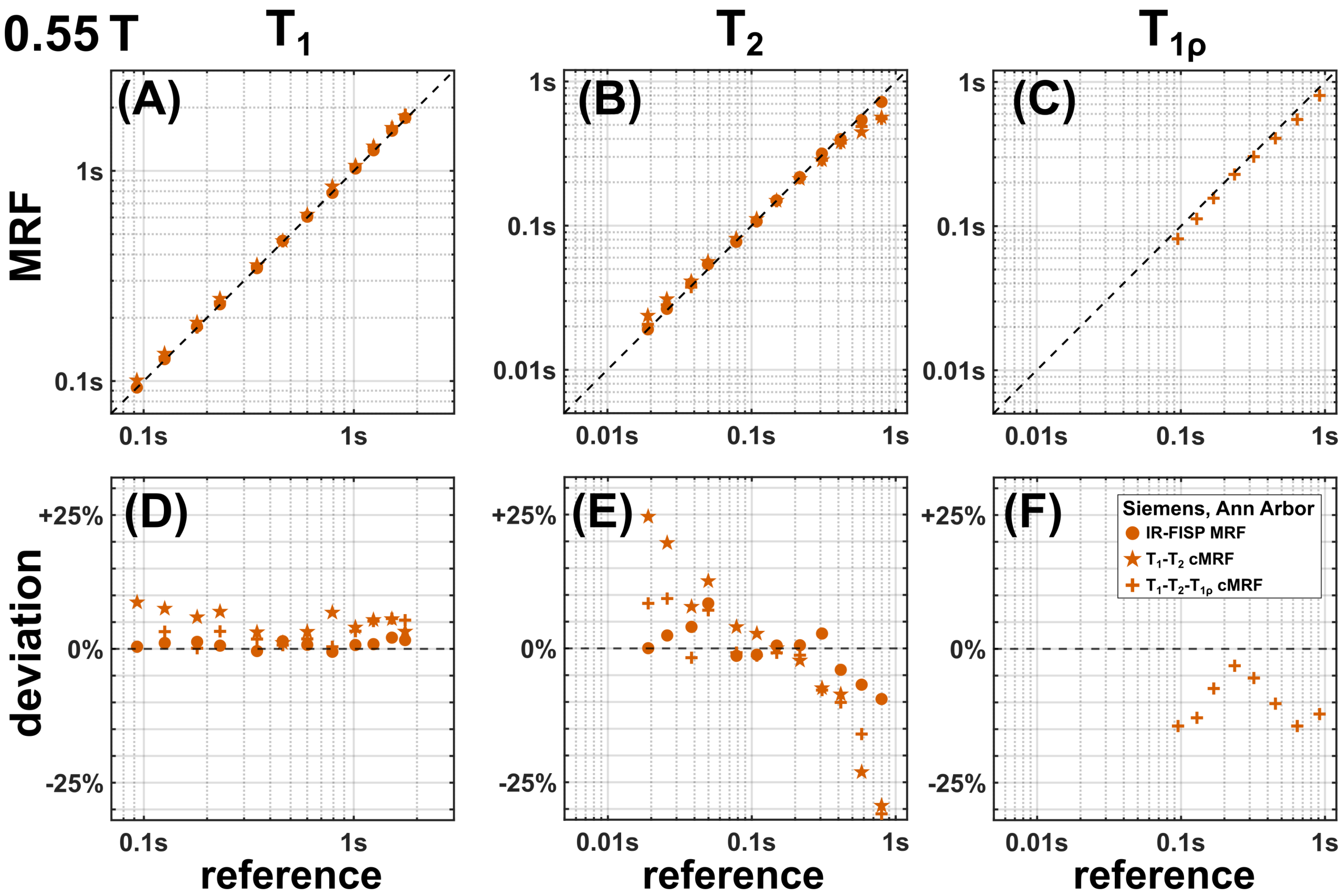

Supplementary Figure S12) Quantitative comparison of T_1, T_2, and $T_{1\rho}$ estimates obtained with three MRF sequences (IR-FISP MRF, T_1–T_2 cMRF, and T_1–T_2–$T_{1\rho}$ cMRF) in the ISMRM/NIST system phantom at 0.55 T. All measurements were acquired on a Siemens Free.Max scanner at the University of Michigan, Ann Arbor. A–C) Estimated relaxation times plotted against the gold-standard reference values for T_1, T_2, and $T_{1\rho}$, respectively. D–F) Corresponding percentage deviations relative to the reference measurements. T_1 estimates remain within a small deviation range across sequences, T_2 estimates exhibit larger variability, and $T_{1\rho}$ estimates show a slight bias toward smaller values.

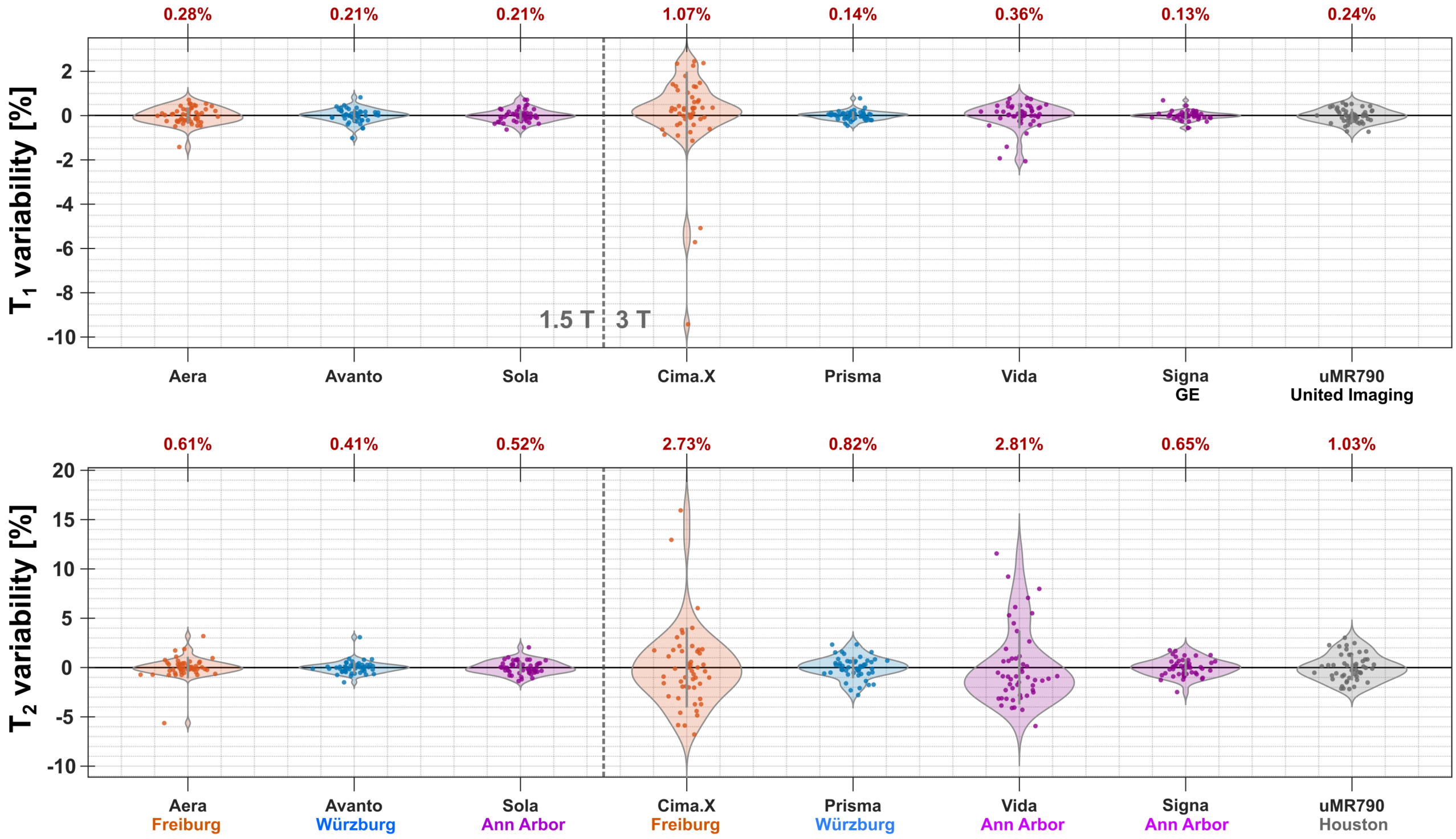

Supplementary Figure S13) Violin plots with overlaid scatter points show the distribution of relative deviations for T_1 (top) and T_2 (bottom) measurements across eight MRI platforms at 1.5 T and 3 T. Data were acquired using the IR-FISP MRF sequence. For each platform, five repeated measurements were performed on the ISMRM/NIST system phantom, and regions of interest corresponding to spheres #3–12 were evaluated. For each sphere and platform, a reference value was defined as the mean across the five repetitions. Relative deviations were then calculated for each measurement as the percentage difference from this platform-specific reference. All deviations were pooled across repetitions and spheres to form the distributions shown for each platform. The reported variability (red labels) corresponds to the mean absolute deviation. Platforms are grouped by field strength (1.5 T and 3 T, separated by a dashed line) and color-coded by acquisition site. The global mean variability for T_1 was 0.23% at 1.5 T and 0.39% at 3 T, and for T_2 0.51% at 1.5 T and 1.61% at 3 T. These results demonstrate high intra-platform repeatability of the IR-FISP MRF sequence across the evaluated MRI systems, with increased variability observed for selected platforms, particularly for T_2 at 3 T.

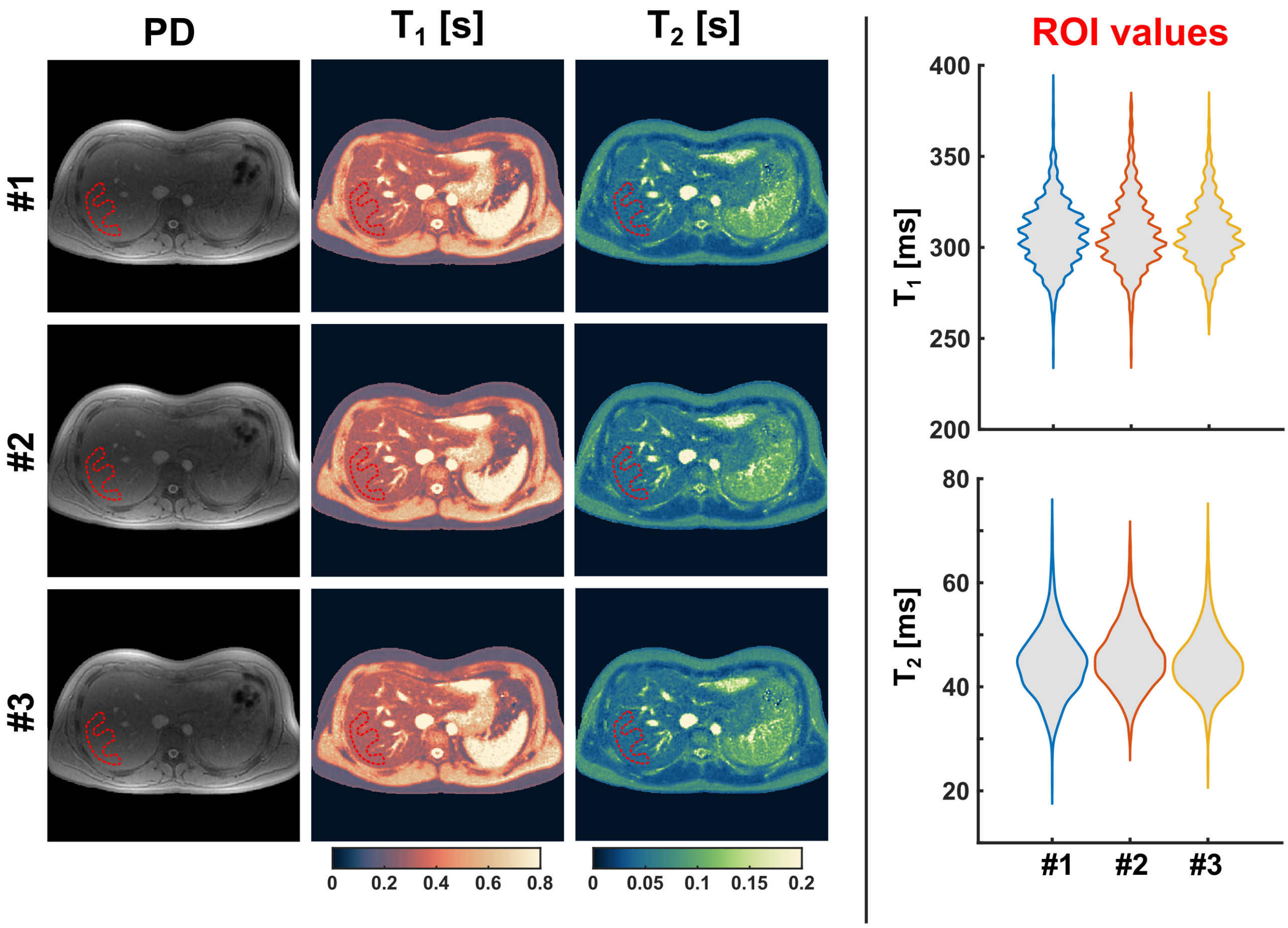

Supplementary Figure S14) Repeatability of *in vivo* T_1 and T_2 quantification using the cardiac MRF sequence without ECG triggering for liver imaging at 0.55 T. Three consecutive breath-hold acquisitions were performed in the same healthy volunteer on a Siemens Free.Max scanner. Representative proton density (PD), T_1, and T_2 maps are shown for each measurement. The red dashed contour indicates the manually defined liver region of interest (ROI) used for quantitative evaluation. Violin plots summarize the distribution of T_1 and T_2 values within the ROI for all three measurements. The estimated relaxation times were highly consistent across repeated acquisitions, with T_1 = 306.7 $\pm$ 17.3 ms, 307.6 $\pm$ 19.3 ms, and 307.6 $\pm$ 17.1 ms, and T_2 = 44.5 $\pm$ 6.3 ms, 45.7 $\pm$ 5.9 ms, and 44.8 $\pm$ 5.8 ms (mean $\pm$ standard deviation within the ROI), demonstrating good repeatability of the OpenMRF framework for *in vivo* quantitative liver imaging in consecutive scans.
